# Development of a serum-based microRNA panel for Alzheimer's disease diagnosis

**DOI:** 10.1515/jtim-2026-0038

**Published:** 2026-06-13

**Authors:** Qi Qin, Xinyi Xia, Xinyu Zhang, Songtao Yang, Guodong Zhao, Chang Su, Yi Tang

**Affiliations:** Department of Neurology & Innovation Center for Neurological Disorders, Xuanwu Hospital, Capital Medical University, National Center for Neurological Disorders, Beijing, China; Miracle (Shenzhen) Biotechnology Co., Ltd., Shenzhen, Guangdong Province, China; School of Chinese Materia Medica, Beijing University of Chinese Medicine, Beijing, China; Neurodegenerative Laboratory of Ministry of Education of the People’s Republic of China, Beijing, China

**Keywords:** Alzheimer's disease, biomarker, diagnosis, microRNAs, serum

## Abstract

**Background and Objectives:**

MicroRNAs (miRNAs) are emerging as promising blood-based biomarkers for Alzheimer's disease (AD) because of their stability and regulatory roles in disease-related pathways. This study aimed to develop a serum-based miRNA panel for AD diagnosis.

**Methods:**

Serum samples from 550 participants were categorized into discovery (85 AD, 65 healthy controls [HCs]), training (73 AD, 53 HCs), and validation (99 AD, 99 HCs, 36 vascular cognitive impairment [VCI], 40 dementia with Lewy bodies [DLB]) cohorts. Through small RNA sequencing and qPCR validation, we identified key miRNAs and constructed a diagnostic panel *via* machine learning.

**Results:**

The 7-miRNA panel achieved area under the curve (AUC) values of 0.970 and 0.928 in the training and validation cohorts, respectively. The panel effectively differentiated AD from VCI (AUC = 0.951) and DLB (AUC = 0.851). Diagnostic performance remained robust across subgroups defined by age, gender, disease severity, and comorbidities (AUC 0.758–0.988). The risk score derived from the 7-miRNA panel was significantly associated with cognitive impairment (Mini-Mental State Examination [MMSE] score, *r* = -0.72) and plasma amyloid pathology biomarkers (Aβ42/40 ratio, *r* = -0.25; p-tau217, *r* = 0.36).

**Conclusions:**

A serum-based 7-miRNA panel, which offers a minimally invasive and accessible approach, has strong diagnostic potential for AD.

## Introduction

Alzheimer’s disease (AD) is the leading cause of dementia, accounting for 60%–80% of cases, and poses a significant global health and socioeconomic burden.^[1]^ Currently, over 55 million people worldwide are affected by AD, and this number is projected to exceed 152 million by 2050 because of the aging global population.^[2]^ With advancements in disease-modifying therapies targeting amyloid and tau pathologies, early and accurate diagnosis has become critical for optimizing therapeutic interventions, monitoring disease progression, and stratifying patients in clinical studies.^[3]^

Current diagnostic methods for AD primarily focus on amyloid-beta (Aβ) and tau biomarkers, which are detected through cerebrospinal fluid (CSF) assays or positron emission tomography (PET) imaging. These methods are considered the gold standard for confirming AD pathology.^[4]^ However, their invasive nature, high cost, and limited accessibility significantly hinder their use in large-scale screening and routine clinical practice. Consequently, there is an urgent need for minimally invasive and widely accessible biomarkers that can reliably detect AD pathology.

MicroRNAs (miRNAs), small noncoding RNA molecules that are typically 19–25 nucleotides in length,^[5]^ regulate gene expression post-transcriptionally,^[6,7]^ and play essential roles in neuroinflammation, synaptic function, and apoptosis—all processes implicated in AD pathogenesis.^[8,9]^ Numerous studies have demonstrated that specific miRNAs are dysregulated in AD, with certain miRNAs influencing Aβ metabolism and tau hyperphosphorylation,^[10, 11, 12, 13]^ the two hallmark processes of AD pathology. Notably, miRNAs exhibit exceptional stability in blood, resist degradation by RNases,^[14,15]^ and maintain their integrity under challenging conditions.^[16,17]^ These features, combined with the relatively simple instrumentation and rapid profiling required for miRNA assays compared with high-sensitivity protein tests,^[18]^ underscore their potential for large-scale screening and clinical applications.^[19,20]^

Prior studies have shown that specific miRNAs have potential to differentiate AD from healthy controls (HCs). A machine learning model based on 21 selected circulating miRNAs achieved an area under the curve (AUC) value of 0.88 for distinguishing AD patients from HCs, but was limited to a small, predefined set of miRNAs.^[21]^ Another study based on the Alzheimer’s Disease Neuroimaging Initiative cohort using three miRNAs could differentiate patients with mild cognitive dementia or AD from HCs, though with modest performance.^[22]^ A recent meta-analysis further summarized 28 consistently dysregulated miRNAs, yet most lacked information on disease severity and inclusion of non-AD patients.^[23]^ These findings highlight the need for rigorous stepwise selection for candidate miRNAs and validation in high-quality cohorts for differential diagnosis of AD from other cognitive impairments.

In light of this, this study aimed to develop and validate a serum-based miRNA panel as a diagnostic tool for AD. Through small RNA sequencing and quantitative real-time polymerase chain reaction (qPCR) validation, we identified key miRNAs and constructed a diagnostic panel *via* machine learning. We further assessed the panel’s ability to differentiate AD from other cognitive impairments, investigated its correlation with neuropsychological scores and established blood biomarkers of amyloid pathology, including plasma phosphorylated tau 217 (p-tau217) and the Aβ42/40 ratio, to evaluate its potential clinical utility.

## Materials and methods

### Study design

**Figure 1:**
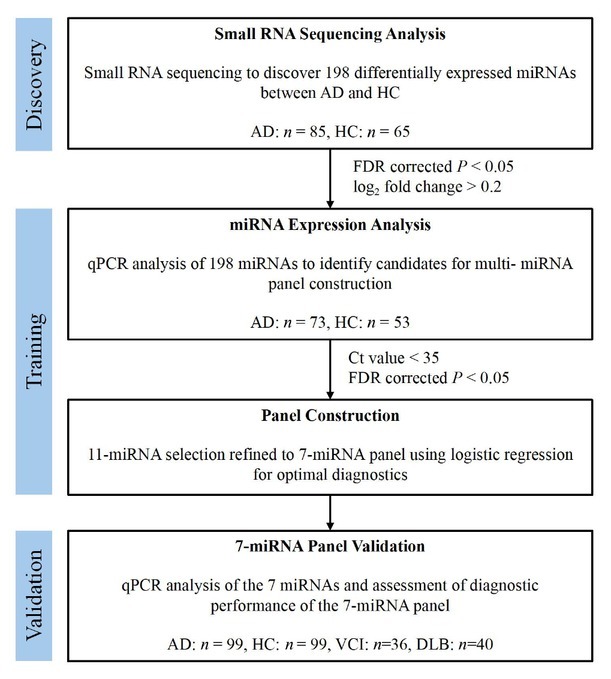
Flowchart of the study design.

This retrospective, single-center study comprised three phases: discovery, training, and validation (Figure 1). A stepwise miRNA screening strategy was applied to identify candidate biomarkers. Sample sizes for each phase were informed by prior literature on AD biomarker studies. In the discovery phase, serum samples from 150 participants (85 AD, 65 HCs) were analyzed *via* small RNA sequencing (Illumina NextSeq 2000) to comprehensively identify differentially expressed miRNAs. In the training phase, the differentially expressed miRNA candidates were detected by qPCR in an independent cohort comprising 126 participants (73 AD, 53 HCs), applying cycle threshold (Ct) values < 35. A logistic regression model was constructed to optimize miRNA selection, and cross-validation was used to reduce overfitting. In the validation cohort, the multi-miRNA panel was validated in 274 participants (99 AD, 99 HCs, 36 vascular cognitive impairment [VCI], 40 dementia with Lewy bodies [DLB]) to evaluate its differential diagnostic performance and correlations with cognitive function and plasma biomarkers. A multicenter validation of this miRNA panel is ongoing, as detailed in the Supplementary File. This study adhered to the Declaration of Helsinki and was approved by the Ethics Committee of Xuanwu Hospital, Capital Medical University (No. 2022201). All experimental procedures were performed under blinded conditions, with laboratory personnel remaining unaware of participants’ clinical diagnoses during the discovery, training, and validation phases.

### Participants

This study included AD, VCI, DLB, and HC participants. Written informed consent was obtained from every participant for the use of serum samples. All participants provided demographic information and underwent cognitive function assessment using the Mini-Mental State Examination (MMSE). Patients with AD, VCI, and DLB were recruited and the diagnosis was made by the consensus of a multidisciplinary team. AD patients were diagnosed in accordance with established diagnostic guidelines,^[24]^ with AD patients confirmed through visual reading of [18F] florbetapir (AV45)-PET imaging and/ or a low Aβ42/40 ratio (<0.10) in CSF.^[25]^ Patients with a clinical diagnosis of probable DLB according to the consensus criteria of McKeith were enrolled.^[26]^ The diagnostic criteria for VCI have been described elsewhere.^[27]^ For AD and DLB patients, those with moderate-to-severe vascular pathology (modified Fazekas score of 2 or 3) were excluded, while only individuals with mild or no vascular pathology (modified Fazekas score of 0 or 1) were included. Cognitive severity among patients (AD, VCI, and DLB) was classified into three categories according to MMSE scores: mild (21–30), moderate (11–20), and severe (≤ 10). HCs were recruited from nearby communities, scored between 27 and 30 on the MMSE, and confirmed to be free from cognitive and neurological symptoms by a physician’s evaluation. Individuals with subjective cognitive complaints or any history of neurological disorders were excluded from the HC group.

Exclusion criteria included major psychiatric diagnoses (*e.g*., major depression or anxiety), other neurological disorders or conditions that can lead to cognitive decline (*e.g*., encephalitis, thyroid dysfunction, or terminal illness), and any factors preventing completion of the study protocol.

### MiRNA expression profiling

#### Blood sample collection and serum/ plasma preparation

For plasma preparation, venous blood was drawn into Ethylenediaminetetraacetic acid (EDTA)-coated tubes to prevent clotting. The blood samples were centrifuged at 1500×*g* for 10 min at 4 °C. The supernatant was collected for a second centrifugation at 12,000×*g* for 10 min at 4 °C to remove any residual cellular debris. The plasma was aliquoted into microcentrifuge tubes and stored at -80 °C until further analysis.

For serum preparation, blood samples were collected in clot activator tubes (Greiner, Cat. No. 45607) and allowed to clot at room temperature for 30 min. Clotted blood was then centrifuged at 2000×*g* for 10 min at 4 °C. The clear supernatant was carefully collected as serum and aliquoted into RNase-free tubes, followed by storage at -80 °C until RNA analysis. All serum samples were collected between March 2023 and March 2024 following standardized procedures to ensure consistency across participants. Hemolysis in serum samples was assessed by visual inspection.^[25]^ Samples exhibiting visible pink or red coloration were considered hemolyzed and excluded. Additionally, quantitative hemolysis assessment was performed by measuring absorbance at optical density (OD) 414 nm for all serum samples. Two samples with abnormally high absorbance values ( > 8-fold above the acceptable range) were excluded, reducing the total sample size from 552 to 550 (detailed quality control metrics are provided in Supplementary Table S1 and Supplementary Figure S1).

#### MiRNA extraction

A total of 200 μL of each serum sample was used for total RNA extraction using the Serum/ Plasma miRNA Extraction Kit (Miracle, Cat. No. MRE00101). The extraction process followed the manufacturer’s protocol, with elution of the extracted RNA in 60 μL of RNase-free water. Quality control was performed by assessing the optical density ratio at 260/280 nm using NanoDrop (Thermo Fisher Scientific). Only samples with a ratio between 1.8–2.2 were included. Otherwise, re-extraction was performed.

To minimize inter-batch variation, all RNA extractions and qPCR assays were conducted in randomized batches containing balanced numbers of AD and control samples. Experiments were performed by the same trained technician using identical reagent lots and instruments to ensure technical consistency. Each qPCR reaction was run in triplicate, and the mean coefficient of variation (CV) for Ct values across replicates was < 5%.

#### Library preparation and next-generation sequencing

Extracted RNA was used for next-generation sequencing library construction with a QIAseq miRNA Next-generation Sequencing (NGS) 96 Index IL kit (Qiagen, Cat. No. 331565) for indexing, alongside a QIAseq miRNA Library Kit (Qiagen, Cat. No. 331505). Library preparation involved several steps: 5 μL of RNA was used for 3’ ligation, with the addition of 1 μL of a spike-in control (QIAseq miRNA Library QC Spike-in 96, Qiagen, Cat. No. 331535; 1:100 dilution). Following the user manual, reverse transcription was carried out with 2 μL of reverse transcription (RT) initiator. After cDNA was cleaned with QIAseq miRNA QMN beads (Qiagen, Cat. no. 333923), the samples were amplified *via* 22 cycles of HT plate indices on a Thermocycler. The amplified samples were cleaned, and their concentrations were measured using Qubit and Bioanalyzer profiling. Single-end 50 bp (SE50) sequencing was conducted on an Illumina NextSeq 2000.

#### NGS data analysis

MiRNA sequencing data preprocessing and analyses were performed with ACGT101-miR V4.2 (LC Sciences, Houston, TX, USA). In brief, adaptors were trimmed using FastQC (V0.11.5) and the raw reads were filtered to remove: (1) reads with more than 10% “*N*” bases; (2) reads with polyA/T/G/C stretches; (3) reads contaminated with 5’ adapter sequences; (4) reads lacking a 3’ adapter or insert. Only those with a base length of 18–26 nucleotides were retained. ribosomal RNA (rRNA), transfer ribonucleic acid (tRNA), small nuclear RNA (snRNA), small nucleolar RNA (snoRNA), messenger RNA (mRNA) and other repetitive sequences were further removed by aligning the reads against the Ensembl_v107 mRNA, RFam and Repbase databases. This yielded a final valid dataset with a minimum of 4.8 million reads per sample. As a final step, miRNAs were identified by aligning reads to human miRNA precursor and mature sequences in the miRBase (v22.1) by Basic Local Alignment Search Tool (BLAST) search, including both known miRNAs and novel 3p- and 5p-derived miRNAs.

#### MiRNA differential expression analysis by qPCR

Serum miRNA was reverse-transcribed using the 5 × miRNA Stem-loop cDNA Synthesis Kit with specific RT primers (Miracle, Cat. No. MR00301) according to the manufacturer’s protocol. The final concentration of the RT primers was 0.1 μmol/L. The RT reaction was performed with 2 μL of isolated miRNA on a SLAN^®^-96S Thermal Cycler (Shanghai Hongshi Medical Technology Co., Ltd.). qPCR amplification was performed on cDNA samples using the 2 × miRNA Stem-loop qPCR Master Mix reagent (Miracle, Cat. No. MR00101) according to the manufacturer’s instructions. Each sample had three replicates. Amplification was conducted on the SLAN^®^-96S Thermal Cycler using a three-step cycling protocol. The relative expression of each miRNA was calculated using the comparative Ct (ΔCt) method, with U6 used as the endogenous control for normalization.

### Establishment of logistic regression model for the serum miRNA panel and calculation of risk score

We developed a binary classifier using an L2-regularized logistic regression model to predict the AD status. ΔCt values of the selected miRNA were used as input features and used to calculate a risk score for each sample *via* a linear model as,


$$\text{Risk Score}=\sum_{i=1}^{7}\beta_{i}*C_{i}+\beta_{0},$$

where *β_i_* and *C_i_* (*i* ∈ [1,7]) denote the trained coefficient and the observed ΔCt value for miRNA *i*, respectively, and denotes the intercept. Higher scores indicate an increased risk of AD. The “glmnet” R package (version 4.1–10) was employed to perform the logistic regression.

Based on the 11 candidate miRNAs selected through the two stages of differential expression analyses, the sequential forward floating search (SFFS)^[28]^ algorithm was used to select the most relevant miRNA biomarkers without performing an exhaustive combination search. Starting with an empty set, SFFS iteratively added the next best miRNA marker to the existing panel, optimizing selection based on AUC of receiver operating characteristic (ROC) curve.

To identify the optimal regularization strength, we conducted a hyperparameter search *via* 5-fold cross-validation exclusively within the training cohort from the discovery dataset, evaluating a logarithmic range of λ values and selecting the value that maximized the average cross-validation AUC-ROC. Five hundred rounds of 5-fold cross-validation were performed. The entire model development process, including feature selection and hyperparameter optimization, was rigorously confined to the training cohort. The final model was refitted on the entire training cohort using the optimal λ. Its performance was subsequently evaluated on the fully independent, held-out validation cohort, ensuring that no validation data were involved in any stage of model development.

### Measurement of plasma biomarkers

For AD patients in the validation cohort, the blood levels of p-tau217 (Cat. No. 81472), Aβ42 (Cat. No. 81301), Aβ40 (Cat. No. 81298), neurofilament light chain (NfL, Cat. No. 81215), Pan-ApoE (Cat. No. 81449), and ApoE4 (Cat. No. 81454) were measured using Lumipulse G Plasma Immunoreaction kits from Fujirebio. The Aβ42/40 ratio was then calculated. Following the manufacturer’s instructions, plasma samples were screened to ensure eligibility based on the following criteria: p-tau217 > 0.217, Aβ42/40 ≤ 0.084, and age > 55 years (*n* = 63). Plasma NfL was measured as a biomarker reflecting the degree of neuronal damage and neurodegeneration. ApoE ε4 carrier status was determined by the ApoE4/Pan-ApoE ratio, where a ratio < 5% was classified as non-carrier and ≥ 5% as carrier.

### MiRNA target prediction and functional annotation

The miRWalk database (http://mirwalk.umm.uniheidelberg.de/) with the random forest-based software TarPmiR was used to predict the target genes of the 7 miRNAs in the panel. Target genes with target scores > 90 were selected. The enrichr database (http://amp.pharm.mssm.edu/Enrichr/) was used for Gene Ontology (GO) functional annotation and Kyoto Encyclopedia of Genes and Genomes (KEGG) pathway enrichment analysis. The GO terms were categorized into biological process (BP), cellular component (CC), and molecular function (MF) categories. KEGG pathway enrichment analysis was conducted to annotate candidate genes, with *P* values calculated using Fisher’s exact test.

### Statistical analysis

The normality of the data distributions was assessed with the Shapiro-Wilk test. Group differences were assessed using *t* tests and the Mann-Whitney *U* test. Categorical variables were assessed using the chi-square test. To analyze the relationships between variables, Pearson and Spearman correlation analyses were used based on the distribution normality. ROC curves and AUC values were used to evaluate the performance of the miRNAs, and the constructed panel was used to differentiate AD from the other groups. Differences in AUCs were assessed using DeLong test. Benjamini-Hochberg false discovery rate (FDR) correction was used for miRNA differential expression analyses. ROC curves were computed from the fixed logistic‑regression risk score, AUCs with 95% confidence intervals (CI) were estimated *via* 1000 bootstrap replicates, and group proportions were compared using chi‑square or Fisher’s exact tests. Subgroup analyses were conducted by age (<65, 65–71, ≥71 years), gender, cognitive severity based on MMSE scores, and comorbidities, including hypertension, hyperlipidemia, and diabetes. The log_₂_-transformed miRNA expression levels were used to calculate *z* scores, and differentially expressed miRNAs were visualized *via* a heatmap using Ward clustering and Euclidean distance metrics. Data analysis and visualization were performed using R (version 4.3.2) with the “ggplot2” package or Python (version 3.11.7).

## Results

### Participants

A total of 550 serum samples were included in the study and were divided into discovery (85 AD, 65 HCs), training (73 AD, 53 HCs), and validation (99 AD, 99 HCs, 36 VCI, 40 DLB) cohorts. Baseline characteristics, ApoE carrier status, comorbidities, and MMSE scores of cohorts are summarized in Table 1. In the discovery cohort, the gender distribution was comparable between the AD patients and HCs (*P* = 0.149), while a significant difference was observed in age (*P* < 0.001). The training cohort showed no significant differences in gender (*P* = 1.0) or age (*P* = 0.11), suggesting well-balanced demographic characteristics between the groups. In the validation cohort including patients with AD, VCI, DLB, and HC, individuals with AD were significantly older than HCs (*P* < 0.001). Significant gender differences were observed between AD and DLB and between VCI and DLB (both *P* < 0.001), accompanied by an additional age difference between AD and DLB (*P* < 0.001). In the training and validation cohorts, AD and HC participants showed a significant difference of ApoE ε4 carrier proportion (*P* < 0.001). For comorbidities, significant differences between diagnostic groups were observed for diabetes in the training cohort (*P <* 0.001) and for hypertension in the validation cohort (*P* = 0.04). Moreover, MMSE scores differed significantly among the three disease groups, with all pairwise comparisons reaching statistical significance (*P* < 0.001, Supplementary Table S2).

**Table 1:** Baseline characteristics of participants in the three cohorts

Characteristic	Discovery cohort (*n* = 150)			Training cohort (*n* = 126)			Validation cohort (*n* = 274)				
	AD (*n* = 85)	HC (*n* = 65)	corrected FDR *P*	AD (*n* = 73)	HC (*n* = 53)	FDR corrected *P*	AD (*n* = 99)	VCI (*n* = 36)	DLB (*n* = 40)	HC (*n* = 99)	FDR corrected *P*
Age, year	65.67 (7.83)	70.03 (4.76)	< 0.001	66.18 (7.11)	67.81 (8.73)	0.27	65.69 (8.14)	65.27 (13.30)	71.18 (6.47)	69.85 (4.86)	< 0.001
Male/Female, *n*	27 / 58	29 / 36	0.15	22 / 51	16/37	1.00	32 / 67	12/24	28/12	43 / 56	< 0.001
MMSE, score	15.49 (5.60)	27 (0)	< 0.001	20.07 (5.58)	27.89 (2.05)	< 0.001	15.61 (5.49)	26.25 (3.95)	18.95 (6.68)	27 (0)	< 0.001
ApoE ε4 carrier, %	/	/	/	62.16%	12.50%	< 0.001	57.78%	/	/	12.50%	< 0.001
Diabetes, %	/	/	/	17.81%	60.38%	< 0.001	15.15%	30.56%	27.50%	23.23%	0.08
Hyperlipidemia, %	/	/	/	65.75%	47.17%	0.06	63.63%	44.44%	47.50%	60.61 %	0.07
Hypertension, %	/	/	/	35.62%	24.53%	0.26	31.31 %	52.78%	30.00%	64.65%	0.04
Education level, n (%)*			< 0.01			0.13					<0.01
1	0 (0)	0 (0)		2 (2.74)	0 (0)		0 (0)	0 (0)	1 (2.50)	0 (0)	
2	17 (20.00)	1 (1.54)		8 (10.96)	0 (0)		19 (19.19)	4(11.11)	4 (10.00)	1 (1.01)	
3	26 (30.59)	24 (36.92)		17 (23.29)	20 (37.74)		27 (27.27)	7 (19.44)	10 (25.00)	30 (30.30)	
4	17 (20.00)	15 (23.08)		26 (35.61)	16 (30.19)		20 (20.20)	11 (30.56)	10 (25.00)	30 (30.30)	
5	15 (17.65)	25 (38.46)		9 (12.33)	17 (32.07)		20 (20.20)	11 (30.56)	6 (15.00)	37 (37.37)	
6	2 (2.35)	0 (0)		3 (4.11)	0 (0)		2 (2.02)	0 (0)	0 (0)	0 (0)	
Missing value	8 (9.41)			8 (10.96)			11 (11.11)	3 (8.33)	9 (22.50)	1 (1.01)	

Age and MMSE scores are presented as mean (SD). Student's *t*-test, Mann-Whitney *U* test, or Kruskal test was used to assess group differences for continuous variables. Chi-square test was used to assess group differences for categorical variables. *Education level: 1- No formal education, 2- Primary school, 3- Middle school, 4- High school, 5-Undergraduate 6- Graduate school. AD: Alzheimer's disease; HC: healthy control; VCI: vascular cognitive impairment; DLB: dementia with Lewy bodies; FDR: false discovery rate; SD: standard deviation; MMSE: Mini-Mental State Examination.

### Discovery phase: NGS-based miRNA screening

**Figure 2:**
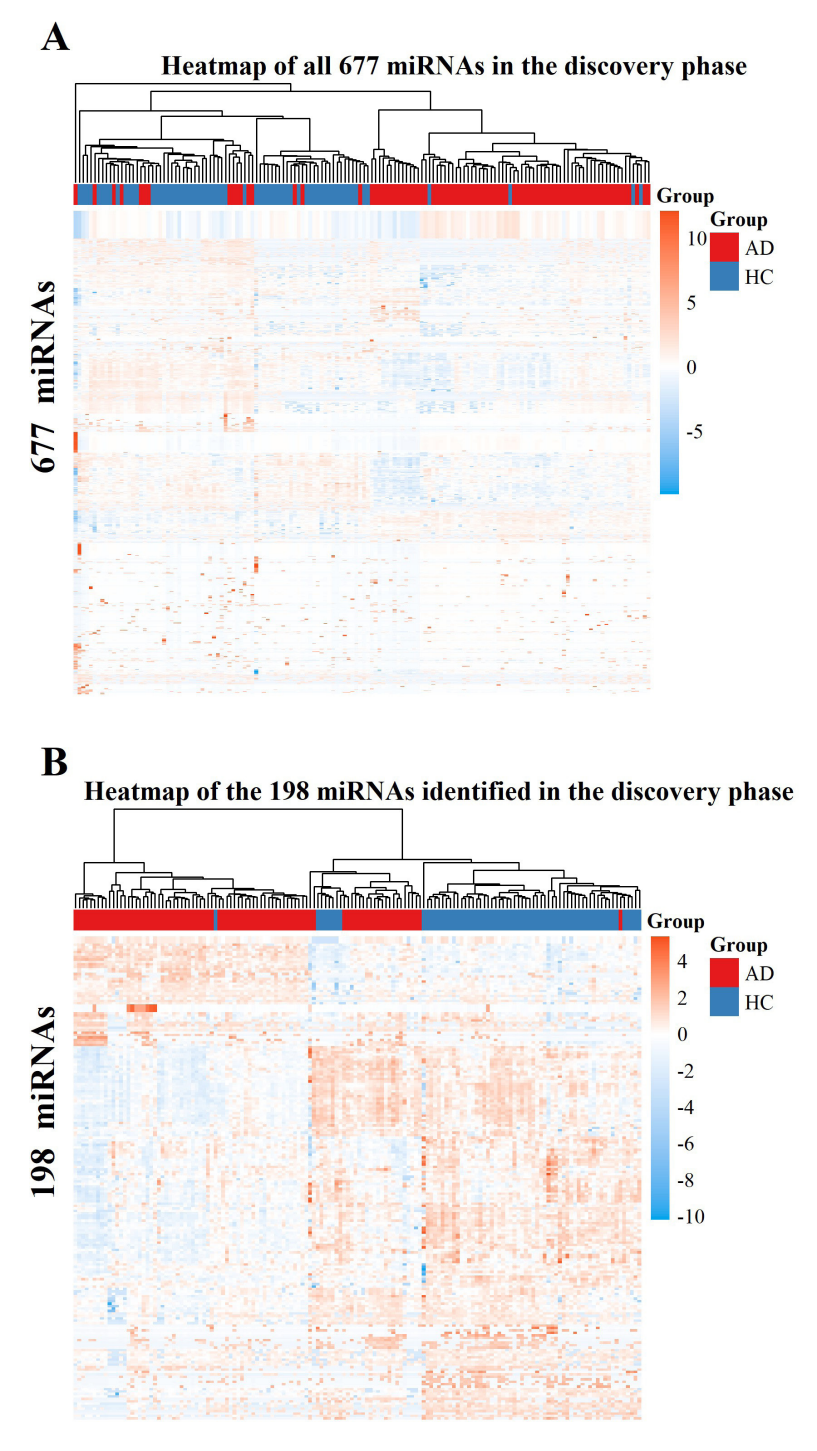
Clustering heatmaps showing 677 detected and 198 differentially expressed serum miRNAs between AD patients and HCs. (A) Heatmap of 677 mature miRNAs identified after sequencing and quality control of serum samples. (B) Heatmap of the 198 miRNAs identified in the discovery phase. AD: Alzheimer’s disease; HC: healthy control.

Sequencing of 150 serum samples yielded an average of 22.4 million total raw reads per sample. After stringent data filtering and quality control to eliminate low-quality and nonspecific reads (average Q20 of 99.3% and Q30 of 97.2%), an average of 11.7 million valid reads per sample were retained. A total of 677 valid mature miRNAs were detected, with an average of 12.7 reads per replicate (Figure 2A).

We applied an FDR-corrected *P <* 0.05 and log_2_ fold change > 0.2 to identify differentially expressed miRNAs, aiming to exclude those with minimal biological relevance. 198 miRNAs were significantly dysregulated between AD patients and controls (Figure 2B, Supplementary Table S3). Of the miRNAs that did not meet these criteria, 475 were excluded based on the FDR threshold and 4 were then excluded based on the fold-change threshold. Among these, 40 miRNAs were upregulated and 145 were downregulated in AD patients compared with HCs (Supplementary Figure S2). Newly identified miRNAs were excluded from further analysis.

### Training phase: miRNA expression analysis via qPCR and panel construction

We performed qPCR to assess the 198 differentially expressed miRNAs in a training cohort comprising 126 subjects (73 AD, 53 HCs). Based on the criteria of Ct values less than 35 and significant differences between AD and HCs, a total of 11 miRNA biomarkers were verified as differentially expressed between the AD and HC groups.

**Figure 3:**
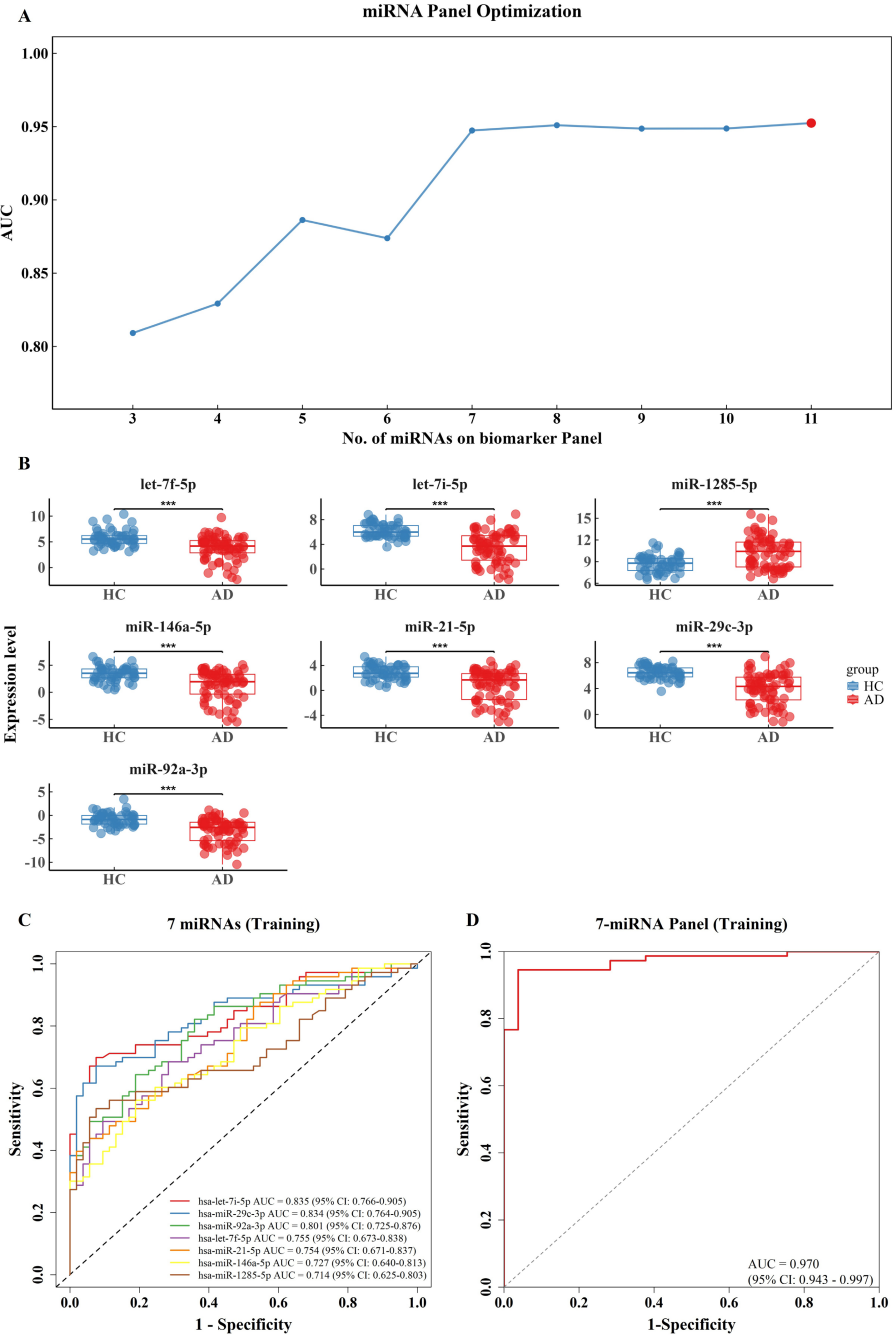
Construction of the 7-miRNA panel for AD diagnosis. (A) AUC of multimiRNA panels comprising 3-11 miRNAs. (B) Boxplots of the ΔCt values of 7 miRNAs in the training cohort. The horizontal line denotes the median value, the box encompasses the upper and lower quartiles, and the whiskers represent the range. (C-D) ROC curves of the 7 individual miRNAs and the 7-miRNA panel in differentiating AD patients from HCs in the training cohort. ^***^P < 0.001. AD: Alzheimer’s disease; HC: healthy control; AUC: area under the curve; ROC: receiver operating characteristic.

Based on 11 candidate biomarkers, a multi-miRNA diagnostic panel was developed and optimized using a logistic regression model with L2 regularization. Cross-validation identified the optimal λ parameter as 1/25. The AUC of the test increased with the number of miRNAs included but reached a plateau at seven, indicating no further performance gain beyond this point (Figure 3A). The final 7-miRNA panel included six upregulated miRNAs (miR-146a-5p, let-7i-5p, miR-21-5p, miR-29c-3p, miR-92a-3p, and let-7f-5p) and one downregulated miRNA (miR-1285-5p) in the AD patients (Figure 3B).

ROC curve analysis revealed that all seven miRNAs effectively distinguished AD patients from HCs, with AUC values ranging from 0.714–0.835. Among these, let-7i-5p demonstrated the highest diagnostic accuracy, with an AUC of 0.835 (95% CI: 0.766–0.905) (Figure 3C). The AUC of the final 7-miRNA panel reached 0.970 (95% CI: 0.943–0.997) in the training cohort (Figure 3D). We further compared the panel with plasma NfL, a biomarker of axonal injury and neurodegeneration, to evaluate its relative diagnostic value of AD. Among 132 available samples, the 7-miRNA panel achieved an AUC of 0.925 for distinguishing AD patients from HCs, significantly outperforming plasma NfL (AUC = 0.607; DeLong test, *P* < 0.001) (Supplementary Figure S3, Supplementary Table S4). The panel’s performance remained robust across subgroups stratified by common comorbidities (diabetes, hyperlipidemia, and hypertension), with AUCs ranging from 0.933 to 0.988 (Supplementary Table S5).

### Validation phase: 7-miRNA panel validation by qPCR

**Figure 4:**
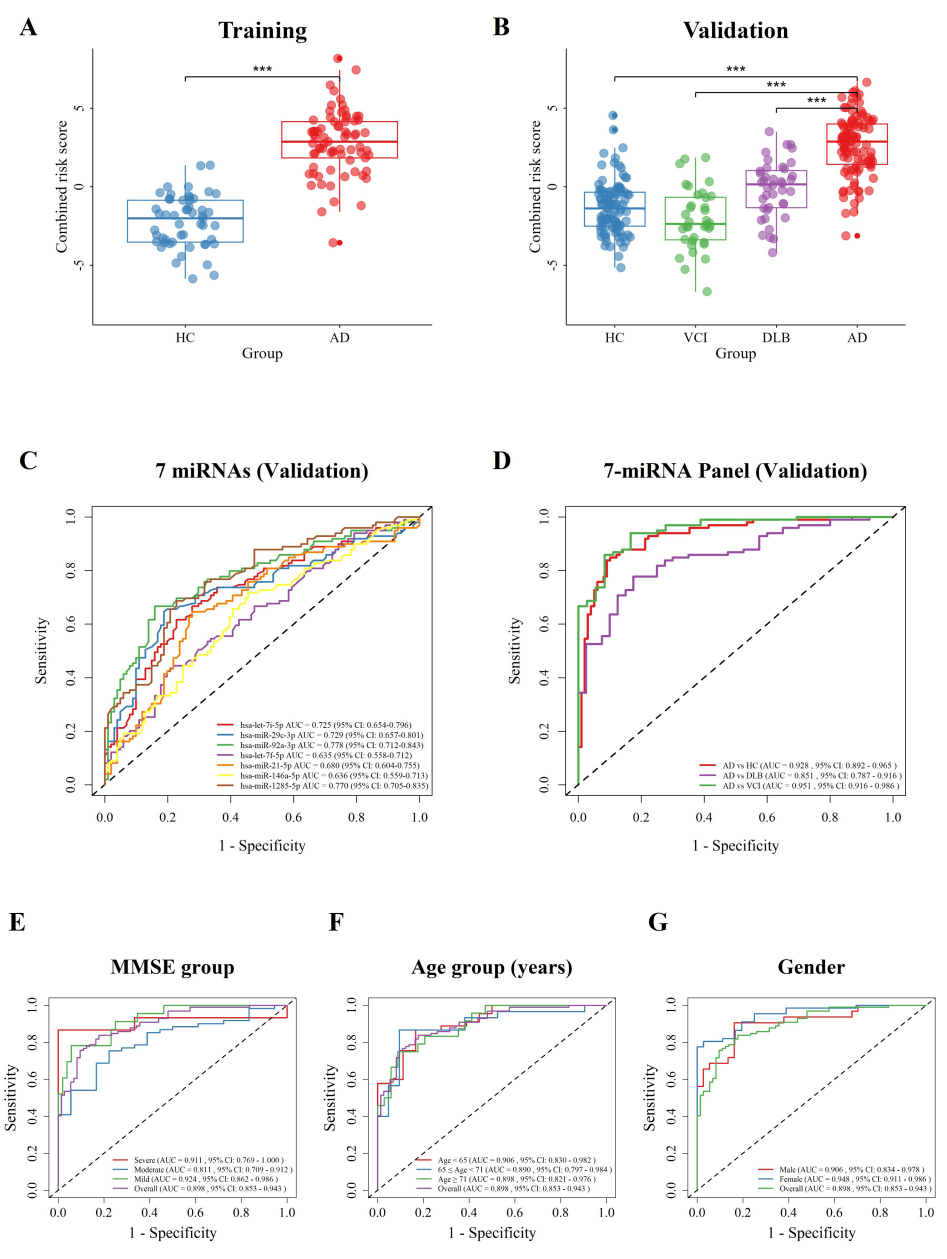
Validation of the 7-miRNA panel for AD diagnosis. (A) Boxplot of the risk scores comparing the AD and HC groups in the training cohort. (B) Boxplot of the risk scores in the validation cohort, including the AD, HC, VCI, and DLB groups. (C) ROC curves of the seven individual miRNAs in differentiating AD patients from HCs in the validation cohort. (D) ROC curves of the 7-miRNA panel in differentiating AD patients from HCs, DLB patients, and VCI patients in the validation cohort. (E-G) ROC curves of the 7-miRNA panel in differentiating AD patients from HCs, stratified by MMSE, age, and gender subgroups. ^***^P < 0.001. AD: Alzheimer’s disease; HC: healthy control; VCI: vascular cognitive impairment; DLB: dementia with Lewy bodies; ROC: receiver operating characteristic; MMSE: Mini-Mental State Examination.

In a validation cohort comprising 274 participants, we further analyzed the differential diagnostic performance of the 7-miRNA panel. A linear risk score was calculated for each sample, with AD patients showing significantly higher risk scores than HCs in both the training and validation cohorts (both *P* < 0.001) (Figure 4A and B). ROC analyses based on qPCR-derived ΔCt values demonstrated that four miRNAs individually discriminated AD patients from HCs with AUCs > 0.7. Among them, miR-92a-3p (AUC = 0.778) and miR-1285-5p (AUC = 0.770) exhibited the highest discriminatory performance (Figure 4C). The 7-miRNA panel’s risk score achieved a high AUC of 0.928 (95% CI = 0.892–0.965) in differentiating AD and HC groups. The panel also effectively differentiated AD from other cognitive impairments, yielding AUC of 0.951 (95% CI = 0.916–0.986) for AD *vs*. VCI and 0.851 (95% CI = 0.787–0.916) for AD *vs*. DLB (Figure 4D), demonstrating strong specificity for AD pathology.

Subgroup analyses further confirmed the robustness of the 7-miRNA panel (Figure 4E-G). Across disease severity subgroups defined by MMSE scores, the panel achieved AUCs of 0.924, 0.811, and 0.911 for mild, moderate, and severe subgroups. Across age and gender subgroups, the panel maintained consistent performance, with AUCs of 0.906, 0.890, and 0.898 for participants aged < 65, 65–71, and ≥ 71 years, respectively, and AUCs of 0.906 and 0.948 for male and female participants. In subgroup analyses stratified by comorbidities, the panel maintained high performance in distinguishing AD patients from HCs (AUC 0.896–0.948), VCI (AUC 0.929–0.974), and DLB (AUC 0.758–0.894) (Supplementary Table S5). Together, these findings demonstrate that the 7-miRNA panel maintains stable and high diagnostic accuracy for AD, independent of demographic or clinical factors.

### Correlations of the levels of the 7 miRNAs with MMSE scores, plasma p-tau217 levels and the Aβ42/40 ratio

**Figure 5:**
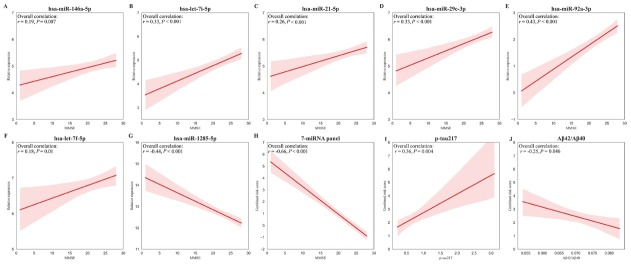
Correlations of the levels of the 7 miRNAs with MMSE scores, plasma p-tau217 levels and the Aβ42/40 ratio. Each plot featured a fitted line representing the overall trend of the data, along with 95% confidence intervals (95% CI). (A-G) Correlations between the MMSE score and the expression of each of the 7 miRNAs. (H) Correlations between the MMSE score and the risk score of the 7-miRNA panel. (I-J) Correlations of the risk score of the 7-miRNA panel with plasma p-tau217 and the Aβ42/40 ratio. MMSE: Mini-Mental State Examination.

We conducted correlation analyses to explore the correlations between miRNA candidates and the whole panel with cognitive function, as indicated by the MMSE score, and other plasma biomarkers controlling for age. In the validation cohort, correlation analyses of the 7 miRNAs and the MMSE score revealed that the 6 upregulated miRNAs (miR-146a-5p, let-7i-5p, miR-21-5p, miR-29c-3p, miR-92a-3p, and let-7f-5p) were positively correlated and that the downregulated miRNA (miR-1285-5p) was negatively correlated with the MMSE score (Figure 5 A-G). The risk score derived from the 7-miRNA panel was negatively correlated with the MMSE score (Figure 5H).

In the AD group in the validation cohort, the plasma level of p-tau217 was 0.75 ± 0.55 ng/L, and the Aβ42/40 ratio was 0.056 ± 0.018. Our correlation analyses revealed that the risk score of the 7-miRNA panel was positively correlated with the level of plasma p-tau217 (*r* = 0.36, *P* = 0.008) but negatively correlated with the Aβ42/40 ratio (*r* = -0.25, *P* = 0.046) (Figure 5I-J).

### Identification of miRNA target genes and their functions in disease pathways

**Figure 6:**
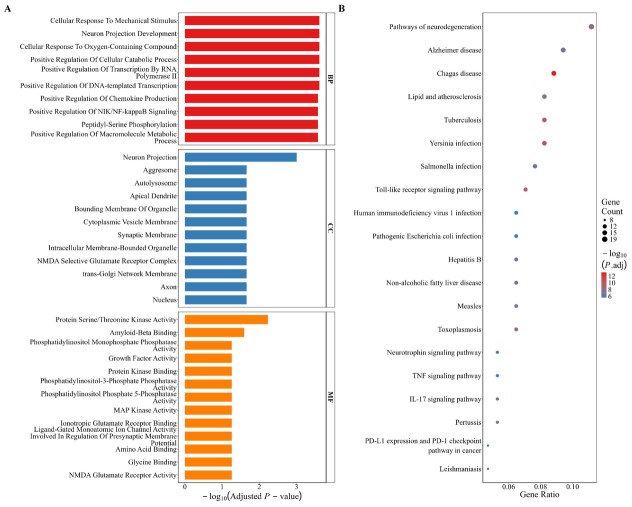
GO and KEGG pathway enrichment analyses of the putative genes targeted by the 7 miRNAs. (A) GO 2023 analysis of the top enriched terms for Biological Process (BP), Cellular Component (CC), and Molecular Function (MF). (B) KEGG 2021 analysis of the top 20 enriched pathways. Adjusted *P* values are log-transformed. GO: Gene Ontology; KEGG: Kyoto Encyclopedia of Genes and Genomes.

Target gene prediction for the 7 miRNA candidates identified 144 putative target genes. The top terms with the lowest adjusted *P* values (*P* < 0.05) for each of the GO categories—BP, CC, and MF—are presented in Figure 6A. The results revealed that the potential target genes were related to neuronal function and structure, such as Aβ binding (GO: 0001540, *P* < 0.001), neuron projection (GO: 0043005, *P* < 0.001), and neuron projection development (GO: 0031175, *P <* 0.001). Other enriched terms included protein serine/threonine kinase activity (GO: 0004674, *P* < 0.001), nucleus (GO: 0005634, *P* < 0.001), and cellular response to oxygen-containing compounds (GO: 1901701, *P <* 0.001).

KEGG pathway enrichment analysis revealed the top 20 enriched pathways, as depicted in Figure 6B. Notable pathways among these pathways included the neurodegeneration (*P* < 0.001), AD (*P* < 0.001), and neurotrophin signaling pathways (*P* < 0.001). Other enriched pathways included inflammation and immune-related pathways, such as Chagas disease (*P* < 0.001), Yersinia infection (*P <* 0.001), Toll-like receptor signaling pathway (*P* < 0.001), and the IL-17 signaling pathway (*P* < 0.001).

## Discussion

With increasing emphasis on early diagnosis and disease monitoring in AD, blood-derived miRNAs have emerged as promising biomarkers because of their stability and accessibility. Following a structured discovery and training phase, this study has developed a 7-miRNA panel comprising miR-146a-5p, let-7i-5p, miR-21-5p, miR-29c-3p, miR-92a-3p, let-7f-5p, and miR-1285-5p. This miRNA panel was successfully validated in a separate cohort, demonstrating its capacity to distinguish AD from VCI and DLB. Bioinformatics analyses linked the 7 miRNAs to genes and pathways implicated in AD pathophysiology, including neurodegeneration, Aβ binding, and neuroinflammatory responses.

The 7-miRNA panel demonstrated high diagnostic accuracy between AD patients and HCs, achieving an AUC of 0.970 in the training cohort and 0.928 in the validation cohort. The 7-miRNA panel also effectively differentiated AD from VCI (AUC = 0.951) and DLB (AUC = 0.851). Its diagnostic performance remained consistent across subgroups stratified by age, gender, disease severity, and presence of comorbidities, supporting the robustness and broad applicability of the panel. These results are comparable to established CSF biomarkers and blood protein biomarkers, underscoring its potential as a diagnostic tool for AD.^[29,30]^ Notably, a prior study reported a 12-miRNA panel in whole blood that achieved 93.3% accuracy in distinguishing AD patients from HCs.^[20]^ Additionally, given the emerging role of plasma NfL as a biomarker for monitoring disease progression and prognosis in AD,^[31]^ we conducted a head-to-head comparison between the 7-miRNA panel and plasma NfL. The 7-miRNA panel demonstrated superior performance in differentiating AD patients from HCs, suggesting its greater specificity in capturing AD-related molecular alterations. These findings highlight the promise of blood-based miRNA profiling as a minimally invasive and scalable approach, particularly in settings where CSF analysis or PET imaging is less accessible or practical. The stability of miRNAs in circulation and the minimally invasive nature of blood collection further increase their appeal for widespread application.

The 7-miRNA panel-derived risk score showed a strong correlation with cognitive impairment severity, as reflected by MMSE scores, and demonstrated significant associations with established AD biomarkers, such as plasma p-tau217 and the Aβ42/40 ratio. The correlation between the 7-miRNA risk score and MMSE was stronger than that with Aβ42/40, suggesting that these miRNAs may represent downstream molecular events associated with neuronal injury and inflammation rather than early amyloid accumulation. Consistent with this interpretation, target gene enrichment analysis indicated that the miRNAs converge on biological pathways involved in synaptic transmission, axonal maintenance, neuroinflammatory signaling, and apoptosis. These processes are well known to contribute to neurodegeneration and cognitive impairment,^[32,33]^ supporting the notion that the 7-miRNA panel reflects the downstream consequences of AD pathology that are more directly linked to clinical severity. These results suggest that the panel not only captures the molecular hallmarks of AD but also reflects clinical manifestations, offering a bridge between the pathological and symptomatic aspects of the disease.

Among the seven miRNAs in the panel, five have been previously studied in the context of blood-based AD biomarkers. Our findings that miR-92a-3p,^[34]^ miR-146a-5p,^[35]^ miR-21-5p,^[36]^ and let-7f-5p ^[37]^ are upregulated in AD patients compared with HCs are consistent with prior studies. However, conflicting results have been reported in the literature,^[20,38,39,40]^ highlighting the complexity of miRNA regulation. For example, while our study revealed the upregulation of let-7i-5p in AD patients, previous studies reported contrasting results.^[41]^ These discrepancies likely stem from the intricate regulation of miRNAs and variability in their expression across plasma, extracellular vesicles, and cerebrospinal fluid.^[42,43]^

The biological relevance of the 7 miRNAs further supports their diagnostic potential. As post-transcriptional regulators, these miRNAs are involved in key pathways implicated in AD, including amyloid precursor protein processing, tau hyperphosphorylation, and neuroinflammatory responses.^[44, 45, 46]^ miR-146a-5p and miR-21-5p are known to regulate the NF-κB pathway,^[47,48]^ which plays a central role in neuroinflammation and neurodegeneration.^[49,50]^ In particular, miR-146a-5p influences microglial phagocytosis and Aβ clearance,^[51]^ making it a potential therapeutic target.^[52]^ Similarly, miR-21-5p, traditionally linked to carcinogenesis, has recently been associated with amyloid pathology and cognitive decline.^[36,50,53]^

The let-7 family has also been implicated in neuroinflammation and immune dysregulation of the nervous system.^[54,55]^ Dysregulation of let-7 expression contributes to neurodegeneration through Toll-like receptor 7 activation.^[56]^ Notably, let-7f-5p has overlapping roles in major depressive disorder and Parkinson’s disease,^[57,58]^ suggesting its involvement in broader neurodegenerative mechanisms rather than AD-specific pathology. miR-92a-3p, another upregulated miRNA in the panel, has been linked to working memory performance and progression from mild cognitive impairment to AD.^[34,59,60]^ Through the miR-92a-CPEB3 axis, it exerts neuroprotective effects by mitigating inflammatory neurodegeneration,^[61]^ although its elevation in frontotemporal dementia indicates a role as a more general marker of neurodegeneration.^[38,62]^

Few studies have examined miR-29c-3p and miR-1285-5p as AD biomarkers. miR-29c-3p has been reported to be significantly downregulated in CSF from AD patients,^[63]^ with associations with CSF Aβ42, sTREM2, and BACE1 activity, which highlights its role in early AD pathogenesis.^[39]^ Furthermore, the miR-29 family has been implicated in motor function,^[50]^ broadening its potential relevance in neurodegenerative processes. miR-1285-5p, previously studied in conditions such as diabetes mellitus,^[64]^ hepatitis B virus infection,^[65]^ and breast cancer,^[66]^ is reported here for the first time in the context of AD. These findings emphasize the novelty of our panel and its potential to identify previously unexplored aspects of AD pathophysiology.

The strengths of this study lie in its multi-phase design, progressing from discovery to validation, and its integration of miRNA profiles with established AD biomarkers. The panel exhibited consistent diagnostic performance across age, gender, comorbidity, and disease severity subgroups, underscoring its potential for early-stage detection. The integration of amyloid pathology-validated cohorts adds to the robustness of our findings. The inclusion of patients diagnosed with other degenerative cognitive impairments further promoted the efficacy of this panel. However, this study has several limitations. As a single-center study, external and multi-center validation is needed to confirm generalizability. Although disease severity subgroups were defined using MMSE scores, the absence of Clinical Dementia Rating scores limits the standard assessment of cognitive status. Incomplete ApoE ε4 data and unconfirmed amyloid negativity in non-AD participants may affect the confidence in the panel’s specificity. Additionally, the cohort may not fully reflect racial or regional diversity, as the current study was conducted exclusively in the Han Chinese population. Future studies incorporating diverse populations, longitudinal follow-up, comparison with various neurodegenerative diseases, and cost-effectiveness evaluation will further strengthen the panel’s clinical relevance.

This study presents a serum-based 7-miRNA panel as a promising, minimally invasive biomarker for AD diagnosis. The panel demonstrated strong diagnostic and discriminative potential, correlated with established AD biomarkers, and bridged molecular pathology with cognitive decline. Future research should employ longitudinal designs to validate its ability to predict disease progression and differentiate AD from other conditions.

## Supplementary Material

Supplementary Material Details
