# Supplementary material for: Development of a serum-based microRNA panel for Alzheimer's disease diagnosis
Source: J Transl Int Med. 2026 Jun 13;14(3):456–69. doi: 10.1515/jtim-2026-0038 (PMC13320530; doi:10.1515/jtim-2026-0038)
Supplement: Supplementary file 1 — Supplementary Material Details [file jtim-2026-0038_sm.pdf]

Supplementary materials

Supplementary Figures

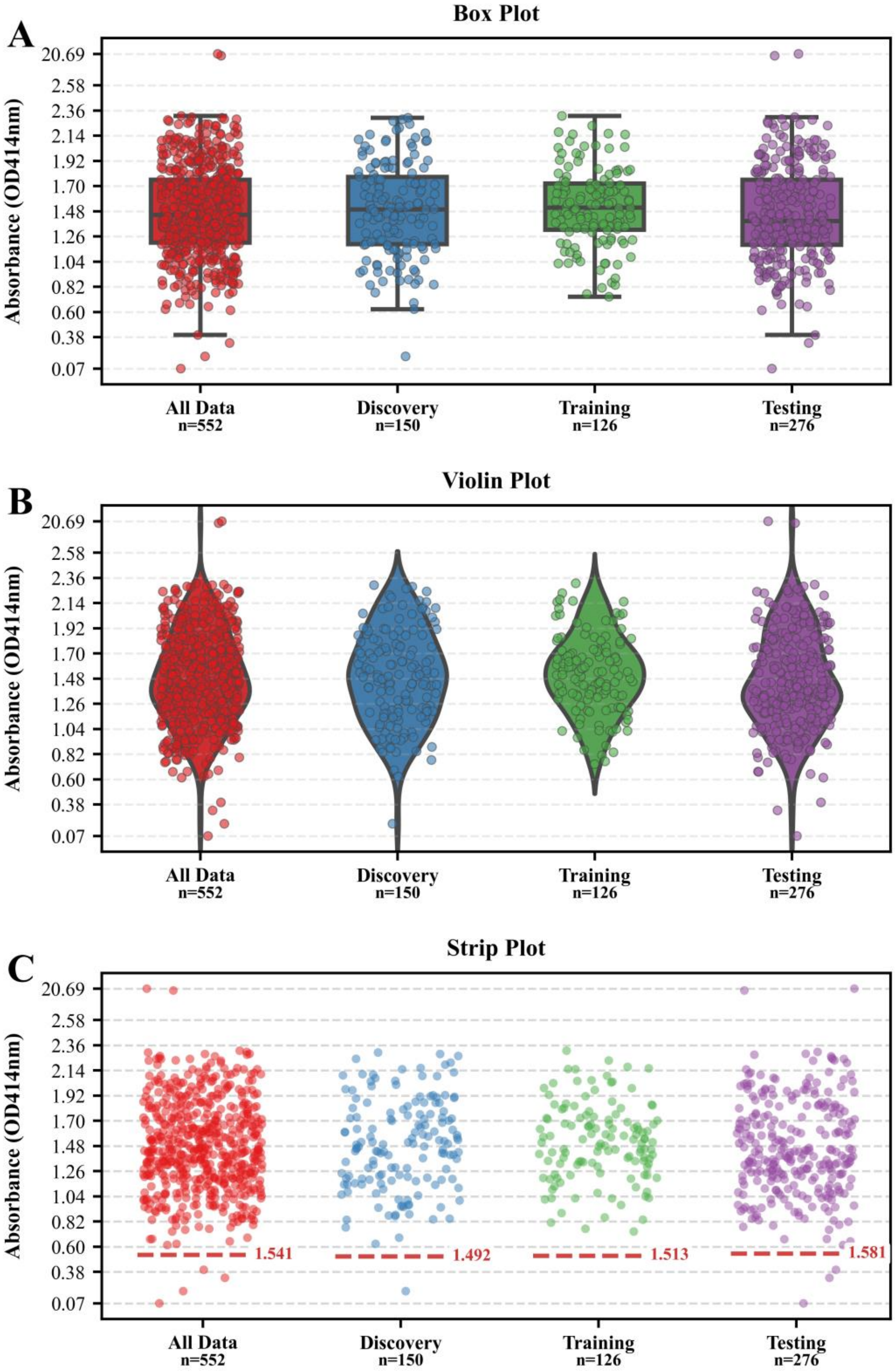

Supplementary Figure S1: Distribution of serum absorbance at OD_{414} nm across all

study cohorts as part of hemolysis quality control. Absorbance at 414 nm was measured via NanoDrop spectrophotometry to assess hemolysis across all study cohorts. (A) Box plots, (B) violin plots, and (C) strip plots with mean values (red dashed lines) show the distribution of OD_{414} nm values in discovery (n = 150), training (n = 126), and validation (n = 276) cohorts. Two validation samples with severely elevated absorbance (19.623 and 20.690, > 8-fold threshold) were excluded due to significant hemolysis, yielding a final cohort of 550 participants. Over 95% of samples demonstrated OD_{414} nm < 3.0, confirming acceptable sample quality for miRNA analysis. OD: optical density.

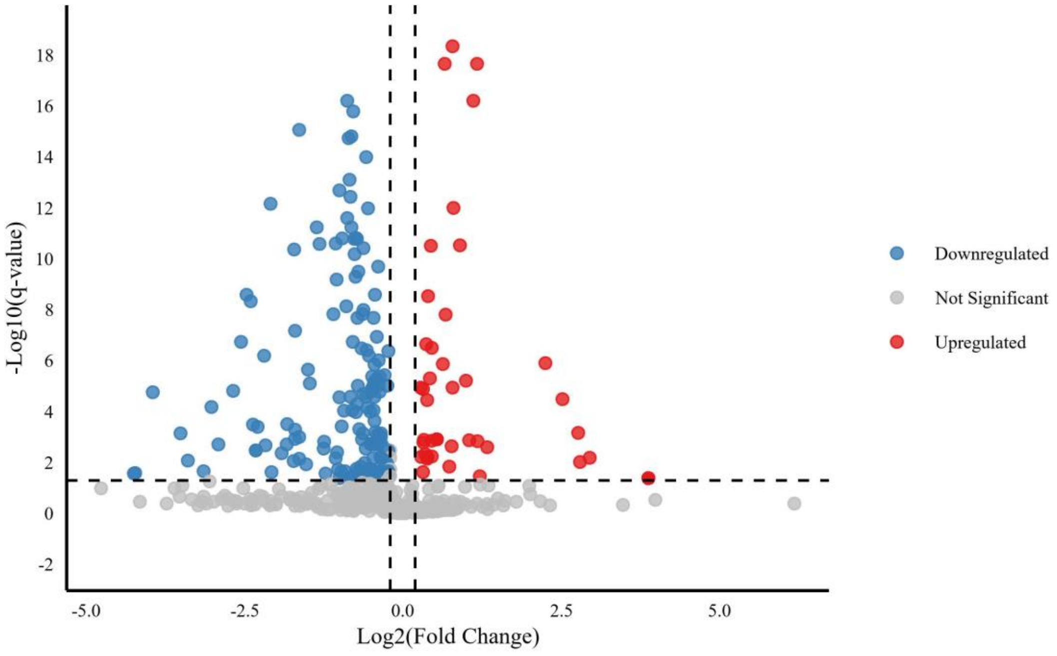

Supplementary Figure S2: Volcano plot of differentially expressed miRNAs. Red and blue colored dots represent miRNAs with an absolute value of $\log_2$ fold change > 0.2 and FDR corrected $P < 0.05$.

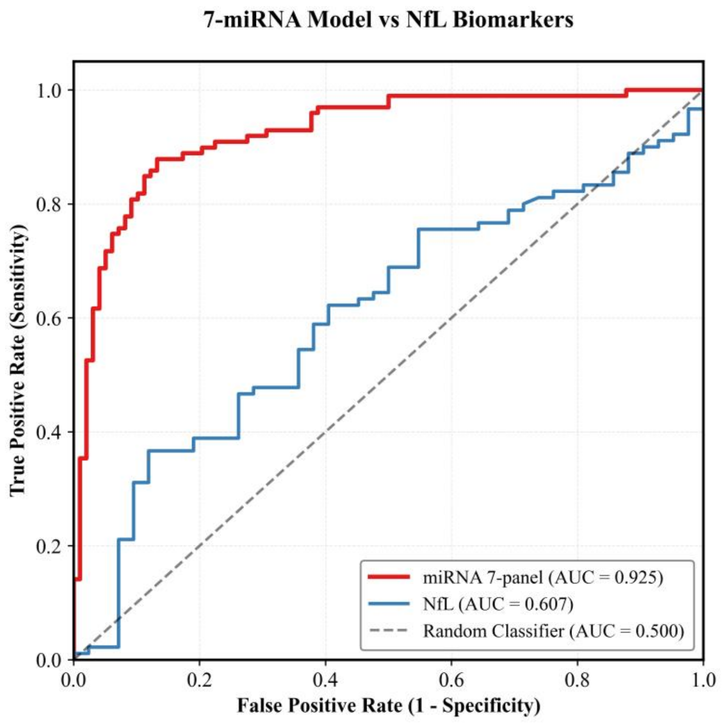

Supplementary Figure S3: Comparative diagnostic performance of 7-miRNA panel and plasma NfL in the validation cohort. The 7-miRNA panel demonstrated significantly superior diagnostic performance (AUC = 0.925, 95% CI: 0.887-0.961) compared to plasma NfL (AUC = 0.607, 95% CI: 0.504-0.706). ROC curves were generated based on paired samples with complete data for both biomarkers (*n* = 132 pairs from the validation cohort). AUC values with 95% CI were calculated using bootstrap resampling (1,000 iterations). Statistical comparison between the two biomarkers was performed using the DeLong's test. AUC: area under the receiver operating characteristic curve; CI: confidence interval; NfL: neurofilament light chain; ROC: receiver operating characteristic.

Supplementary Tables:

Supplementary Table S1: Statistical summary of hemolysis quality control by absorbance measurements.

**Dataset**	***N***	**Mean**	**SD**	**Median**	**Min**	**Max**	**Range**	**Q25**	**Q75**	**IQR**	**CV%**	**Skewness**	**Kurtosis**
All Data	1104	1.541092391	1.189230453	1.449	0.073	20.69	20.617	1.20525	1.75425	0.549	77.16801794	13.9475394	217.0196543
Discovery Data	150	1.491953333	0.403817826	1.4965	0.193	2.295	2.102	1.19275	1.77875	0.586	27.06638453	-0.135056101	-0.299441928
Training Data	126	1.51318254	0.338275282	1.5115	0.734	2.311	1.577	1.31825	1.7205	0.40225	22.35521975	0.00660478	-0.293288548
Validation Data	276	1.580539855	1.640929376	1.3955	0.073	20.69	20.617	1.186	1.75425	0.56825	103.8208161	10.63794895	119.6570187

Absorbance at 414 nm was measured using NanoDrop spectrophotometry (Thermo Fisher Scientific) for all serum samples prior to RNA extraction. The table presents descriptive statistics including sample size (N), mean, standard deviation (SD), median, range (Min-Max), interquartile range (IQR, Q25-Q75), coefficient of variation (CV%), skewness, and kurtosis for each cohort. The "All Data" row represents the initial dataset of 552 samples before quality control exclusions. Two samples from the validation cohort with absorbance values of 19.623 and 20.690 were excluded due to excessive hemolysis, reducing the validation cohort from 276 to 274 samples and the total study population from 552 to 550. The high skewness (13.95) and kurtosis (217.02) in the "All Data" and "Validation Data" rows reflect the presence of these hemolyzed samples with extreme absorbance values. After exclusion, the Discovery, Training, and final Validation cohorts demonstrated acceptable and comparable absorbance distributions (mean: 1.49-1.58, CV: 22-27%), confirming consistent sample quality across study phases. N: number of samples; SD: standard deviation; Min: minimum value; Max: maximum value; Q25: 25th percentile; Q75: 75th percentile; IQR: interquartile range (Q75-Q25), CV%: coefficient of variation (percentage), OD_{414} nm: optical density at 414 nanometers. Statistical calculations for "All Data" and "Validation Data" include the two excluded outlier samples. After exclusion, the final analysis cohorts comprised: Discovery (*n* = 150), Training (*n* = 126), and Validation (*n* = 274), totaling 550 samples.

Supplementary Table S2: Comparisons of MMSE, gender, and age distribution across validation cohort.

Factor	Subgroup	AD	VCI	DLB	Total Count	FDR corrected *P* (AD vs. VCI)	FDR corrected *P* (AD *vs*. DLB)	FDR corrected *P* (VCI *vs*. DLB)
MMSE	Mild	23	34	20	77	< 0.001	2.46e-03	< 0.001
	Moderate	61	2	17	80			
	Severe	15	0	3	18			
Gender	Male	32	12	28	72	1.0	< 0.001	0.009
	Female	67	24	12	103			
Age	≥71 years	21	10	20	51	0.33	5.14e-04	0.11
	65-71 years	28	13	14	55			
	<65 years	50	13	6	69			

Chi-square test was used to assess the overall association between one of the stratification variables (MMSE / gender / age) and diagnosis (AD / VCI / DLB) within each cohort. For the validation cohort, shown p-values were FDRs after adjusting the raw *P*-values for multiple comparisons. AD: Alzheimer's disease; VCI: vascular cognitive impairment; DLB: dementia with Lewy bodies; HC: healthy control; MMSE: Mini-Mental State Examination.

MMSE stratification criteria: Mild (≥21), Moderate (10-20), Severe (<10).

Supplementary Table S3: List of 198 differentially expressed miRNAs with an absolute value of $\log_2$ fold change > 0.2 and FDR-corrected *P* < 0.05.

miR_name	$\log_2$FC	*P*	FDR corrected *P*
hsa-miR-16-2-3p_L+1R-2	0.787781061	6.4973E-22	4.60658E-19
hsa-miR-15b-3p_R-1	0.664826941	6.31094E-21	2.23499E-18
hsa-miR-4732-3p	1.175088008	9.45693E-21	2.23499E-18
hsa-miR-451a_R-2	1.114741396	4.20423E-19	6.27063E-17
hsa-miR-21-3p	-0.875774086	4.42217E-19	6.27063E-17
hsa-let-7g-5p	-0.780700621	1.37891E-18	1.62941E-16
hsa-miR-450b-5p_R-1	-1.631449	8.57561E-18	8.68586E-16
hsa-let-7a-5p	-0.812793616	1.72061E-17	1.52489E-15
hsa-miR-23a-3p_R-2	-0.850320796	2.33115E-17	1.83643E-15
hsa-miR-423-5p	-0.573263676	1.4525E-16	1.02982E-14
hsa-miR-589-5p_R-1	-0.841439265	1.20511E-15	7.76751E-14
hsa-miR-181a-2-3p_R-3	-1.001140989	3.49768E-15	2.06654E-13
hsa-miR-181b-5p_R-2	-0.821792761	6.6785E-15	3.64235E-13
hsa-miR-378f_R+1_1ss14CT	-2.086361172	1.3716E-14	6.94616E-13
hsa-miR-16-5p	0.803284477	2.15015E-14	1.0163E-12
hsa-miR-181a-5p_R-2	-0.545283746	2.33154E-14	1.03316E-12
hsa-miR-548e-3p_R-1	-0.874846775	5.99051E-14	2.49839E-12
hsa-miR-148a-5p_R-2	-1.358112914	1.506E-13	5.75836E-12
hsa-miR-374a-3p	-0.812422845	1.54314E-13	5.75836E-12
hsa-let-7f-5p	-0.733714645	4.25577E-13	1.50867E-11
hsa-miR-223-5p_R+1	-0.958144357	4.69273E-13	1.58435E-11
hsa-miR-30e-3p_1ss22CT	-0.723226054	5.4146E-13	1.68546E-11
hsa-miR-24-3p_R-2	-0.765825374	5.46765E-13	1.68546E-11
hsa-miR-338-5p_R-1	-1.054311312	8.63241E-13	2.55016E-11
hsa-miR-193a-5p	-1.308981134	9.25899E-13	2.62585E-11
hsa-miR-96-5p_R-2	0.907562851	1.09576E-12	2.98805E-11
hsa-miR-92a-3p	0.447262591	1.19876E-12	3.14786E-11
hsa-miR-148a-3p	-0.616721588	1.49838E-12	3.79412E-11
hsa-miR-3130-3p	-1.716034531	1.74387E-12	4.26345E-11
hsa-miR-99b-5p	-0.75530221	2.80354E-12	6.62569E-11
hsa-miR-148b-3p	-0.386815673	9.05581E-12	2.07115E-10
hsa-miR-152-3p	-0.702759722	1.45473E-11	3.22313E-10
hsa-miR-629-5p	-0.745804773	2.33838E-11	5.02398E-10
hsa-miR-144-5p	-1.045377621	3.19314E-11	6.65863E-10
hsa-let-7b-5p	-0.434436493	1.31934E-10	2.59837E-09
hsa-miR-542-3p_R-2	-2.467291869	1.37758E-10	2.63975E-09
hsa-miR-25-3p	0.399604598	1.56279E-10	2.91583E-09
hsa-miR-27a-5p	-2.397097261	2.54924E-10	4.63439E-09
hsa-miR-424-5p_R-1	-0.891717968	4.07889E-10	7.22982E-09
hsa-miR-27a-3p_R-1	-0.62021188	5.8577E-10	1.01295E-08

hsa-miR-423-3p	-0.631806818	9.66356E-10	1.52255E-08
hsa-miR-576-3p_R-1	-1.092260335	9.88714E-10	1.52391E-08
hsa-miR-106a-3p_L+2R-3	0.679709247	1.01772E-09	1.53525E-08
hsa-miR-500a-3p_R-1	-0.456686795	1.44274E-09	2.09254E-08
hsa-miR-10b-5p_R-1	-0.714679539	1.44618E-09	2.09254E-08
hsa-miR-582-3p_R-1	-1.700768271	4.75692E-09	6.61305E-08
hsa-miR-21-5p	-0.410970556	8.56038E-09	1.16717E-07
hsa-miR-150-3p_R-2	-2.549182006	1.37287E-08	1.83654E-07
hsa-miR-30a-3p_R-1	-0.787357127	1.40752E-08	1.84802E-07
hsa-miR-363-3p_R-1	0.369151117	1.85645E-08	2.30916E-07
hsa-miR-425-5p	0.458060419	2.56519E-08	3.13572E-07
hsa-let-7e-5p	-0.6522898	2.8126E-08	3.32355E-07
hsa-miR-651-5p_R-1	-0.565557363	3.42403E-08	3.91555E-07
hsa-miR-128-3p_R-2	-0.223871816	3.71893E-08	4.18527E-07
hsa-miR-320d_R-1	-2.188463349	5.91521E-08	6.53004E-07
hsa-miR-361-3p_R-1	-0.532057273	5.98664E-08	6.53004E-07
hsa-miR-103a-3p	-0.376946274	9.46633E-08	9.727E-07
hsa-miR-624-5p_R-1	2.253373262	1.27412E-07	1.27233E-06
hsa-miR-942-5p_L-2R+1	0.633943972	1.39146E-07	1.3702E-06
hsa-miR-501-3p_R-2	-0.443026331	1.45034E-07	1.40862E-06
hsa-miR-378c_R-5	-1.491773758	2.32284E-07	2.22553E-06
hsa-let-7d-3p_R-2	-0.285223927	3.98889E-07	3.67288E-06
hsa-let-7f-2-3p_1ss22CT	-0.480048334	4.63422E-07	4.21239E-06
hsa-miR-191-5p_R-1	-0.361135309	4.78653E-07	4.29576E-06
hsa-miR-92b-3p_R-2	0.43133214	5.69826E-07	5.05008E-06
hsa-miR-27b-3p	-0.410797871	5.81982E-07	5.09414E-06
hsa-miR-576-5p_R-2	1.002251428	6.96075E-07	6.0185E-06
hsa-miR-361-5p	-0.449068813	8.45603E-07	7.22328E-06
hsa-miR-1304-3p_1ss13CA	-1.46414234	9.47015E-07	7.80737E-06
hsa-miR-2110_R-1	-0.376545699	1.12828E-06	9.1948E-06
hsa-miR-340-5p	-0.459378784	1.184E-06	9.53931E-06
hsa-miR-181a-3p	-0.706245394	1.24257E-06	9.81572E-06
hsa-let-7i-5p_R-1	-0.239321206	1.246E-06	9.81572E-06
hsa-miR-107_R-2	0.283558803	1.46043E-06	1.13785E-05
hsa-miR-3143_R-4	0.787894926	1.5109E-06	1.16438E-05
hsa-miR-30a-5p_R-1	-0.485667018	1.75149E-06	1.30955E-05
hsa-miR-151b_R+2	0.323800235	1.75468E-06	1.30955E-05
hsa-miR-873-5p_L+1R-3	-2.676779581	2.08795E-06	1.54204E-05
hsa-miR-155-5p_R-1	-0.49272156	2.17337E-06	1.58857E-05
hsa-let-7d-5p	-0.362263542	2.29149E-06	1.65783E-05
hsa-miR-618_R-2	-3.944730316	2.4605E-06	1.76211E-05
hsa-miR-142-3p_R-1	-0.621607518	2.90221E-06	2.05767E-05
hsa-miR-221-3p	-0.446959295	3.77537E-06	2.63866E-05
hsa-miR-320a-3p	-0.580108979	3.7961E-06	2.63866E-05

hsa-miR-99a-5p_R-1	-0.820538081	3.88007E-06	2.67084E-05
hsa-miR-1307-5p	-0.99523751	4.01683E-06	2.73839E-05
hsa-miR-4685-3p_R-2	2.521966841	4.90195E-06	3.30999E-05
hsa-miR-486-5p_R-1	0.384213419	5.34879E-06	3.5442E-05
hsa-miR-29a-3p_R-1	-0.72196941	7.91121E-06	5.19356E-05
hsa-miR-125a-3p_R-1	-3.016770785	1.04653E-05	6.74539E-05
hsa-miR-1260a_1ss9TG	-0.552921936	1.14887E-05	7.27276E-05
hsa-miR-3615_R+1	-0.462346686	1.41835E-05	8.8992E-05
hsa-miR-181d-5p	-0.794618663	1.4517E-05	9.02854E-05
hsa-miR-100-5p_R-1	-0.92884212	1.52244E-05	9.38614E-05
hsa-miR-183-5p	-0.510863153	1.66382E-05	0.000101694
hsa-miR-1284_R-1	-0.733927765	1.73078E-05	0.000104882
hsa-miR-146b-5p_R+1	-0.445376089	3.87802E-05	0.00023301
hsa-miR-483-5p	-1.8243555	5.12415E-05	0.000305296
hsa-miR-642a-3p_R-1	-2.365425161	5.39488E-05	0.000318747
hsa-miR-378a-3p	-0.963262305	6.64853E-05	0.000389571
hsa-miR-2355-3p_L-2	-2.289671188	6.80668E-05	0.000393072
hsa-miR-664a-5p_R-2	-0.682321149	8.76679E-05	0.000501262
hsa-miR-320c_R-1	-1.696010908	9.21366E-05	0.000522599
hsa-miR-197-3p	-0.430595966	0.000110725	0.000623047
hsa-miR-296-5p	2.770102542	0.000121295	0.000677151
hsa-let-7i-3p_R-1	-0.340886819	0.000127336	0.00070514
hsa-miR-10a-5p_R-1	-0.629104499	0.000128298	0.00070514
hsa-miR-3605-5p_R-2	-3.508771984	0.000131984	0.000719822
hsa-miR-151b_R+3	-0.401645123	0.000151063	0.000817586
hsa-miR-374a-5p_R-1	-0.367539389	0.00015564	0.000835974
hsa-miR-12136_R+8	-1.631189997	0.000187069	0.000997234
hsa-miR-199b-3p_R-1	-0.34165731	0.000192508	0.001018569
hsa-miR-342-5p	-1.697899362	0.000223032	0.001171329
hsa-miR-95-3p_R-1	-0.639257196	0.000236529	0.001233082
hsa-miR-548d-5p_R-2	0.539758275	0.000246442	0.001257032
hsa-miR-548ay-5p_R-1	0.539758275	0.000246442	0.001257032
hsa-miR-548ae-5p	0.539758275	0.000246442	0.001257032
hsa-miR-584-5p_R-1	-0.364051261	0.000250809	0.001270168
hsa-miR-19a-3p_R-2	0.335363479	0.000255913	0.001286824
hsa-miR-18a-3p_1ss23GA	1.048895737	0.000274184	0.001368988
hsa-miR-503-5p_R-2_1ss21CA	0.459960684	0.000276488	0.00137084
hsa-miR-3688-3p_R-2	1.181634635	0.000292338	0.001429433
hsa-miR-378i_R+1_1ss9AT	-1.240280743	0.000317817	0.001536088
hsa-miR-29c-3p_R-1	-0.419503502	0.000318484	0.001536088
hsa-miR-186-5p	0.330274679	0.000339109	0.001624516
hsa-miR-222-3p_R+4	-0.332329094	0.000391415	0.001862504
hsa-miR-28-3p	-0.506167225	0.000399144	0.00188662
hsa-miR-744-5p_R-1	-0.513417787	0.000402316	0.001889021

hsa-miR-190a-5p_R-1	-2.907093414	0.000418938	0.001954124
hsa-miR-192-5p_R-1	-0.420901918	0.000434138	0.001995328
hsa-miR-450a-5p_R-2	-0.438352065	0.00043551	0.001995328
hsa-miR-378d_R-2	-1.828951495	0.000436214	0.001995328
hsa-miR-23a-5p_L+1R-2	-2.165715583	0.000464204	0.002109747
hsa-miR-1294_R-2	0.776256569	0.000521287	0.002354092
hsa-miR-1306-5p_R-2	1.333556711	0.000566961	0.002544149
hsa-miR-769-5p	-0.581888081	0.000634768	0.002812817
hsa-miR-320b_R-2	-1.24516105	0.000657543	0.00289564
hsa-let-7b-3p_R-1	-0.266631621	0.000680502	0.002978244
hsa-miR-1246_L-2R+1	-2.321354452	0.000764575	0.003303264
hsa-miR-1290_R-1_1ss13TG	-2.321354452	0.000764575	0.003303264
hsa-miR-140-3p_R+1	-0.22340339	0.000825916	0.003527556
hsa-miR-134-5p	-1.037951456	0.000945692	0.004014943
hsa-miR-760_R+2	-1.913176991	0.001044693	0.004408855
hsa-miR-1285-3p_R-2	0.354989397	0.001105795	0.004639105
hsa-miR-421	0.458940405	0.001373435	0.005728032
hsa-miR-345-5p_R+1	-0.347054987	0.001426994	0.005916599
hsa-miR-19b-3p	0.29294095	0.001445632	0.005959028
hsa-miR-1180-3p_R-2	0.390771424	0.001465151	0.006004577
hsa-miR-1255b-5p	2.95203783	0.001650237	0.006669486
hsa-miR-23b-3p_R-5	-0.332961158	0.001655613	0.006669486
hsa-let-7c-5p	-0.463629935	0.001719577	0.006888025
hsa-miR-338-3p_R+1	-1.059133576	0.001734868	0.006910231
hsa-miR-7976_R+1	0.38556046	0.001811429	0.007174876
hsa-miR-6852-5p	-1.629848855	0.001833597	0.007222334
hsa-miR-425-3p_L+1R-2	-0.28500881	0.002158823	0.008456384
hsa-miR-371b-5p	-3.390037854	0.002188228	0.008524471
hsa-miR-99b-3p_R-3	-1.721387248	0.002248686	0.00871212
hsa-miR-5010-5p_R-1	2.800566263	0.002481303	0.009561108
hsa-miR-326_R+1	-0.598874305	0.002733259	0.01047503
hsa-miR-22-5p_R-1	-0.248670824	0.002825118	0.010768863
hsa-miR-671-3p	-1.523365495	0.003077619	0.011668621
hsa-miR-196b-5p_R-1	0.739610983	0.003786119	0.014248184
hsa-miR-29b-3p_R-3	-0.660960692	0.003811738	0.014248184
hsa-miR-1260b_1ss9AG	-0.336210209	0.003818272	0.014248184
hsa-miR-221-5p	-0.528806022	0.00474988	0.017539922
hsa-miR-130b-3p_R-2	-0.72778575	0.004897786	0.017992385
hsa-miR-24-2-5p_L+1R-1	-1.021525775	0.005099033	0.018635124
hsa-miR-4454_L-2	-0.505007429	0.005139606	0.018674593
hsa-miR-185-3p_R-1	-0.493834071	0.005162511	0.018674593
hsa-miR-26b-5p_R+1	-0.227753724	0.006110352	0.02188
hsa-miR-127-3p	-0.909283478	0.006287395	0.022296553
hsa-miR-193b-5p_R-3	-3.140111283	0.006289578	0.022296553

hsa-miR-215-5p_R-1	-0.704703616	0.006330062	0.022328429
hsa-miR-30b-5p	-0.239657756	0.00645595	0.022659747
hsa-miR-22-3p	-0.344969422	0.006534924	0.022789058
hsa-miR-874-3p	-0.96747291	0.006557077	0.022789058
hsa-miR-130b-5p_R+1	-0.355345669	0.006641576	0.022970134
hsa-miR-125b-2-3p_L-2	-2.071564223	0.006969519	0.023987324
hsa-miR-4732-5p	0.323483392	0.007124885	0.024403592
hsa-miR-188-5p_R+1	-4.221974653	0.00766792	0.026137284
hsa-miR-3679-5p_R-4	-4.243415145	0.008069796	0.027375529
hsa-miR-454-3p	-1.219390489	0.008162382	0.027557755
hsa-miR-146a-5p	-0.251949908	0.008423792	0.028305539
hsa-miR-335-5p_R-2	-0.408065882	0.008561068	0.028631118
hsa-miR-150-5p	-0.423849931	0.008708957	0.028988971
hsa-miR-484_R-1	-0.201777547	0.010111361	0.033499788
hsa-miR-4286_R+1	-0.674297567	0.010201551	0.033641393
hsa-miR-499a-5p	-0.450659565	0.010664895	0.03500653
hsa-miR-548a-3p	1.218639503	0.010771706	0.035032749
hsa-miR-122-5p_R-1	-0.591456766	0.01176633	0.037940984
hsa-miR-1307-3p_R+1	-0.494804204	0.011808737	0.037940984
hsa-miR-548l_R-1	-0.812722334	0.011826456	0.037940984
hsa-miR-382-5p	-0.778692895	0.012004337	0.038338175
hsa-miR-3611_R-2	-0.827205153	0.012124013	0.038546751
hsa-miR-548au-5p_L-1_1ss21CA	3.876731011	0.013051405	0.040944453
hsa-miR-548c-5p_L-1R-1_1ss21CA	3.876731011	0.013051405	0.040944453
hsa-miR-548ak_L-1_1ss9CT	3.876731011	0.013051405	0.040944453
hsa-miR-10a-3p_R-1	-0.997370699	0.013438427	0.041972884

Supplementary Table S4: Statistical comparison of diagnostic performance between 7-miRNA panel and plasma NfL.

**Biomarker**	**AUC**	**95% CI**	***P* (DeLong test)**
7-miRNA panel	0.925	0.887-0.961	0.942
NfL	0.607	0.504-0.706	

AUC: area under the receiver operating characteristic curve; CI: confidence interval; NfL: neurofilament light chain.

Supplementary Table S5: Diagnostic performance of the 7-miRNA panel stratified by comorbidities.

Cohort	Comparison	Subgroup	N (AD)	N (HC/DLB/VCI)	AUC (95% CI)
**Training**	AD vs HC	With diabetes	13	32	0.971 (0.904-1.000)
		Without diabetes	60	21	0.933 (0.831-0.996)
		With hyperlipidemia	48	25	0.971 (0.931-1.000)
		Without hyperlipidemia	25	28	0.940 (0.858-0.997)
		With hypertension	26	13	0.988 (0.950-1.000)
		Without hypertension	47	40	0.944 (0.887-0.992)
**Validation**	AD vs HC	With diabetes	15	23	0.896 (0.779-0.982)
		Without diabetes	84	76	0.933 (0.891-0.969)
		With hyperlipidemia	63	60	0.943 (0.896-0.976)
		Without hyperlipidemia	36	39	0.920 (0.853-0.973)
		With hypertension	31	64	0.948 (0.897-0.985)
		Without hypertension	68	35	0.896 (0.824-0.953)
	AD vs VCI	With diabetes	15	11	0.946 (0.821-1.000)
		Without diabetes	84	25	0.947 (0.906-0.981)
		With hyperlipidemia	63	16	0.929 (0.849-0.987)
		Without hyperlipidemia	36	20	0.974 (0.926-1.000)
		With hypertension	31	19	0.951 (0.879-0.996)
		Without hypertension	68	17	0.947 (0.895-0.989)
	AD vs DLB	With diabetes	15	11	0.758 (0.542-0.935)
		Without diabetes	84	29	0.850 (0.776-0.919)
		With hyperlipidemia	63	19	0.819 (0.713-0.913)
		Without hyperlipidemia	36	21	0.894 (0.807-0.963)
		With hypertension	31	12	0.828 (0.677-0.931)
		Without hypertension	68	28	0.849 (0.763-0.922)

1. Multicenter study summary

Table 1: Collaborating institution details.

Center	Institution	Key Study Responsibilities	Ethics Approval ID
01	Xuanwu Hospital, Capital Medical University (Beijing, China)	Participant recruitment, biosample collection, core lab	No. 2024002
02	The First Hospital of Hebei Medical University (Hebei, China)	Participant recruitment, biosample collection	No. 2024808
03	Peking University Shenzhen Hospital (Shenzhen, China)	Participant recruitment, biosample collection	No. 2024072A

2. Recruitment strategy

2.1 Standardization framework

To ensure methodological consistency across all participating centers, a unified operational framework was established prior to the study. This includes the development of a comprehensive Standard Operating Procedure (SOP) manual and harmonized case report forms. These documents standardize all key workflows from participant identification, eligibility screening and informed consent procedures to clinical assessments, biospecimen collection, processing, and data entry. These documents served as binding references for all sites, ensuring protocol adherence, inter-site comparability, and data integrity.

2.2 Participant screening and recruitment

Participant recruitment employed a multi-tiered strategy, combining systematic electronic screening with on-site clinical identification and professional referrals. At each site, a standardized electronic case report form (eCRF) was embedded within the local electronic health record system to screen potentially eligible participants according to the inclusion and exclusion criteria. Concurrently, the clinical research coordinators (CRCs) conducted active case-finding during routine outpatient clinic visits in relevant departments, including neurology, memory clinics, and geriatrics, to further suitable candidates. In parallel, a structured referral network was established involving cooperating neurologists, psychiatrists, and primary care physicians. These referring clinicians receive training on the study eligibility criteria to recruit eligible patients directly following their clinical diagnosis.

2.3 Personnel training and certification

A standardized, tiered training program was conducted across all centers to ensure procedural consistency. Prior to recruitment, all site principal investigators attended an initiation meeting focused on comprehensive protocol review, clarification of operational responsibilities, and discussion of potential logistical challenges. Subsequently, centralized training sessions were delivered either in person or virtually to CRCs and clinical research associates (CRAs). The training curriculum covered Good Clinical Practice (GCP) principles, informed consent procedures, administration of neuropsychological assessments, standardized collection and processing of biospecimen aligned with SOPs and data management protocols. All personnel performing critical study tasks were required to pass written or practical competency assessments before being certified to undertake study-related duties.

2.4 Quality control and recruitment monitoring

The central data coordination center, Xuanwu Hospital, Capital Medical University (Beijing), maintained a secure, web-based monitoring platform. This system enabled real-time oversight of site-specific recruitment progress, screening failure documentation, and data completeness indicators. Monthly teleconferences with investigators were held to review recruitment status, share effective site practices, and address adverse events. Furthermore, periodic protocol compliance audits were conducted centrally. These audits involved a review of randomly selected informed consent forms and eligibility verification documentation to ensure adherence to protocol specifications and regulatory requirements.

3. Sample size

3.1. Sample size estimation

This clinical trial adopts a comparative design to evaluate the performance of the 7-miRNA panel against a clinical reference standard for Alzheimer's disease (AD) diagnosis. The Sample size was calculated using the single-group target value method. The target (minimum acceptable) sensitivity and specificity were set at 80% and 90%, respectively, based on our current study results and clinical performance data from the existing biomarker-based AD assays. The minimum required sample sizes for the AD (n^{+}) and non-AD (n^{-}) groups were determined using the following formulas:

$$n^{+} = \frac{[1.96\sqrt{0.8(1-0.8)} + 0.84\sqrt{0.86(1-0.86)}]^2}{(0.86-0.8)^2}$$

$$n^{-} = \frac{[1.96\sqrt{0.9(1-0.9)} + 0.84\sqrt{0.95(1-0.95)}]^2}{(0.95-0.9)^2}$$

AD group: 322 participants

Non-AD group: 239 participants

To ensure adequate representation across diverse disease subtypes, stages, and severity levels, and accounting for an anticipated 15% exclusion rate, the planned enrollment numbers are as follows:

AD patients: ≥ 379

Non-AD patients: ≥ 282, including minimum quotas for:

- Dementia with Lewy Bodies (DLB): ≥ 40
- Vascular Cognitive Impairment (VCI): ≥ 40
- Parkinson's Disease (PD): ≥ 40

Total sample size: ≥ 661 participants

3.2. Sample size allocation

This multicenter study will be conducted at three qualified clinical trial sites. Recruitment targets will be dynamically guided by each site's capacity and ongoing performance by employing a competitive enrollment strategy. Each site is expected to enroll a minimum of 100 participants, including at least 30 AD patients. Final enrollment numbers per site will be confirmed upon study completion and may be adjusted based on site-specific considerations such as local patient availability and disease epidemiology.
